# Case report: when art is faced with brain surgery: acute change in creative style in a painter after glioma resection

**DOI:** 10.3389/fonc.2024.1394609

**Published:** 2024-03-28

**Authors:** Hugues Duffau

**Affiliations:** ^1^ Department of Neurosurgery, Gui de Chauliac Hospital, Montpellier University Medical Center, Montpellier, France; ^2^ Team “Plasticity of Central Nervous System, Stem Cells and Low-grade gliomas,” INSERM U1191, Institute of Functional Genomics, Montpellier, France; ^3^ University of Montpellier, Montpellier, France

**Keywords:** art, brain surgery, creativity, glioma, case report

## Abstract

**Background:**

Strong interactions between art and health are well-known. While advances in brain surgery resulted in an improved preservation of sensorimotor, visuospatial, language and cognitive functions, creative abilities received less attention. However, creativity may represent a critical issue to resume an optimal quality of life, especially in artists. Here, a unique case of sudden change in creative style in a painter who underwent glioma resection is described. This prompts to explore further creative thinking and its clinical implications in routine practice.

**Methods:**

A 36-year-old right-handed woman experienced inaugural seizures, allowing the discovery of a right frontal lesion. The patient was a professional painter and did not complain about any decline in her creativity. The preoperative neurological examination was normal.

**Results:**

Surgery was achieved with a maximal tumor resection through a frontal lobectomy. A WHO grade II oligodendroglioma was diagnosed. A regular surveillance was performed without adjuvant oncological treatment. The patient did not exhibit postoperative functional deterioration and she returned to normal activities including painting during 15 years. Remarkably, even though her creative activity was judged by the patient herself to be rich and satisfying, her style drastically changed from surrealism and mysticism to cubism whereas she was not able to explain why.

**Conclusion:**

This is the first report of acute modification of the painting style following frontal lobectomy for a low-grade glioma, supporting that brain resective surgery may impact creativity. While neglected for many decades, this complex human ability should be evaluated more regularly in neurosurgical practice, particularly in artists.

## Introduction

Strong links between art and medicine are well-known, as evidenced by a recent WHO review which supported a critical role of the arts in the prevention of illness, promotion of good health, and treatment of acute and chronic diseases arising across the lifespan (1). The value of artistic creativity has especially been observed in brain-damaged patients who have benefited from art-therapy in the context of neurorehabilitation (2). It also seems that cerebral injury, in turn, might have an influence on patient’s creative abilities since many reports have described changes in how they approach and produce art (3). Nonetheless, even though several observations have been published for various brain disorders, such as degenerative diseases (Alzheimer’s and Parkinson’s disease, frontotemporal and Lewy body dementia, or corticobasal degeneration) or stroke, there is currently no report of art-related change after brain resective surgery.

Here, a unique case of acute modification of the creative style in a painter who underwent glioma removal is described. This prompts to explore further creative thinking and its clinical implications in routine practice.

## Case report

**Figure 1:**
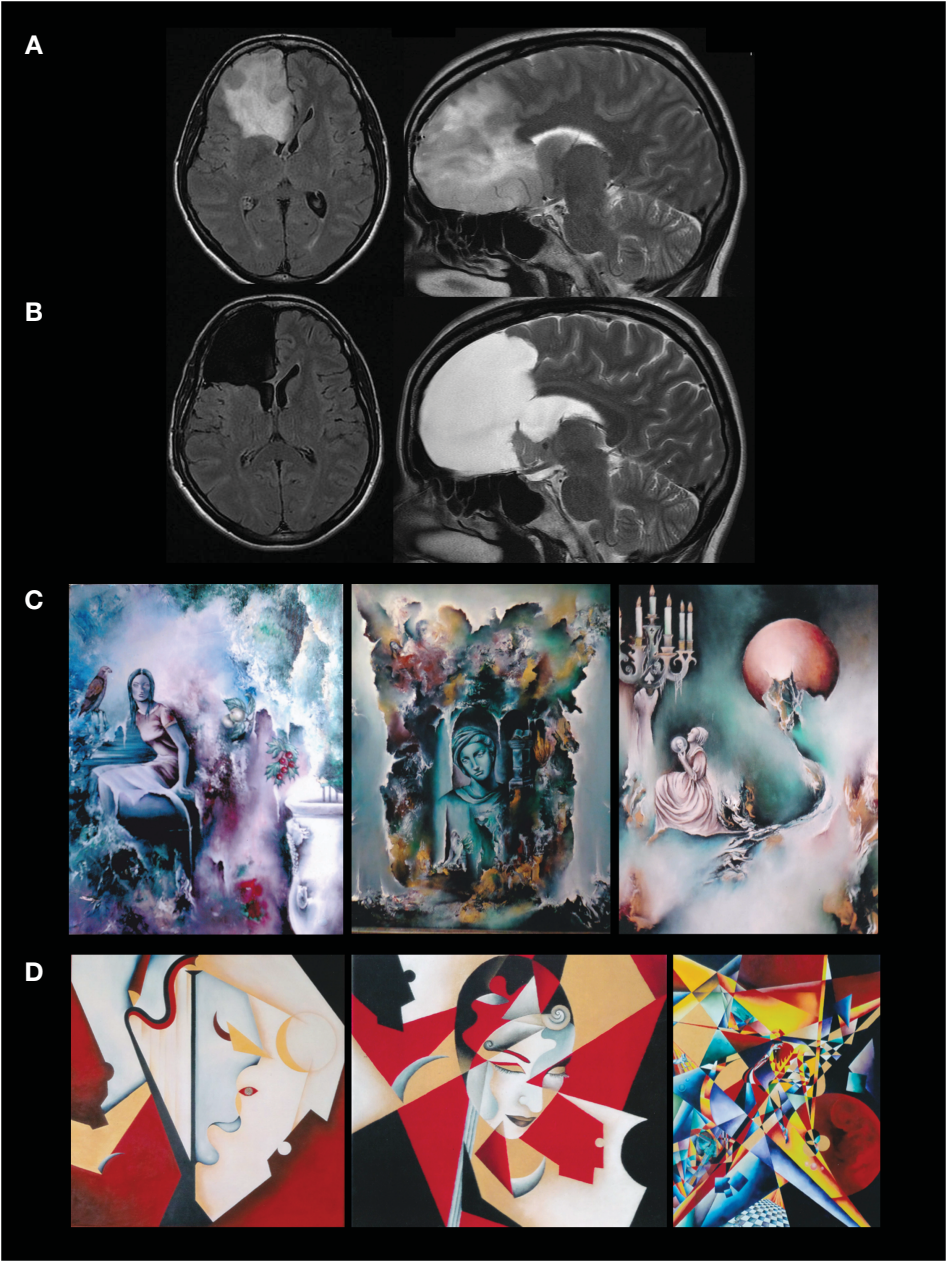
**(A)** preoperative axial FLAIR-weighted MRI (left) and sagittal T2-weighted MRI (right) showing a right frontal hypersignal typical for a low-grade glioma. **(B)** postoperative axial FLAIR-weighted MRI (left) and sagittal T2-weighted MRI (right) showing a right frontal lobectomy involving the DLPFC, ACC and MPFC, with a complete resection of the tumor. **(C)** Three works from her preoperative period. **(D)** Postoperative period illustrating the change in content and technique.

A 36-year-old right-handed woman with no previous medical history experienced inaugural seizures, allowing the discovery of a right frontal lesion. The patient was a professional painter and did not subjectively complain about any decline in her creativity. The preoperative neurological examination was normal. The tumor was voluminous (105mL) and involved the frontal structures, including the medial prefrontal cortex (MPFC), the anterior cingulate cortex (ACC) and the corpus callosum (**Figure 1A**).

Surgery was achieved with a maximal resection of the tumor through a right frontal lobectomy (**Figure 1B**). A WHO grade II oligodendroglioma was diagnosed. A regular surveillance was performed without administration of adjuvant oncological treatment. The patient did not exhibit postoperative functional deterioration and she returned to normal activities including painting for 15 years. Surprisingly, even though her creative activity remained rich and was judged by the patient herself to be satisfying, her style drastically changed - whereas she was not able to explain why - from “surrealism and mysticism” (**Figure 1C**) to “cubism” (as defined by the artist herself) (**Figure 1D**).

The study was approved by an independent institutional review board of the ethical comity of research from the French National College of Neurosurgery (N°00011687–2024/07). Works illustrating the different periods of the patient’s creativity are reproduced with authorization.

## Discussion

Although advances in brain surgery resulted in an improved preservation of sensorimotor, visuospatial, language and higher-order cognitive functions (4), creative abilities have received less attention. However, creativity may represent a critical issue to resume an optimal quality of life, especially in artists. Here, this is the first report of sudden change in artistic style in a painter following large resection of a right frontal glioma, even if the patient was still able to be creative.

Recent developments in neurosciences have led to preliminary hypothesis regarding the neural substrates underpinning artistic activities. From a biochemical perspective, the influence of dopamine agonists in creativity has been evoked. Lhommée et al. (5) described the case of a painter with a Parkinson’s disease who experienced a change in content and technique of painting before and after deep-brain stimulation of the sub-thalamic nucleus. However, due to the role of prefrontal cortex for creativity, the authors did not rule out that the bilateral insertion into frontal lobe of microelectrodes and deep brain stimulation leads had an impact on painting. Indeed, from a connectome perspective, creative cognition has been correlated not only with cortical areas such as the dorsolateral prefrontal cortex (DLPFC) and the ACC, but also with the dynamic interaction across large-scale neural circuits (6, 7). First, the default-mode network (DMN) which mainly consists of the MPFC, posterior cingulate cortex, precuneus and temporoparietal junction, is involved in elaborative processing and self-generated thought, including mind-wandering, mental simulation, social cognition, autobiographical retrieval, and episodic future thinking – as supported by functional imaging (8). Interestingly, a recent series using stereo-electroencephalography in epilepsy patients showed that direct cortical stimulation at the level of several DMN hubs induced a decrease in creative thinking (9). Second, the executive fronto-parietal network (FPN) is also implied in creative cognition (6, 7). This control network is composed of lateral prefrontal (including DLPFC and ACC) as well as anterior inferior parietal regions, and its activity is correlated with cognitive processes which need externally-directed attention, working memory and task-set switching (10). In this integrative framework, an increased interplay between DMN and FPN has been observed during artistic performances, especially visual art (11). Moreover, the salience network which includes anterior insula and cingulate, seems to play an active role in such a DMN/FPN coupling critical for idea generation (6–8).

Whereas art-making changes have been already observed in the event of progressive neurodegeneration, this has not previously been described after brain surgical lobectomy, especially for visual art. In the case reported here, even though functional neuroimaging has not been achieved, one can hypothesize that the massive right frontal resection which involved the DLPFC, ACC and MPFC, thus with a disconnection of a part of the DMN and FPN, might have impacted the artistic style by modulating the balance across brain systems underlying creative thinking. In other words, artistic creativity should be conceived as a multidimensional entity relying on dynamics across neural circuits, in the framework of a meta-networking organization of cerebral processing, i.e., with perpetual succession of new equilibrium states within network of networks (12). By applying this concept to artists who should undergo removal for a brain glioma, it has recently been proposed to achieve awake surgery with intraoperative direct electrostimulation (DES) mapping while the patients are performing on-line multi-tasking throughout the resection into the operating room (13). This monitoring of several functional systems (e.g., sensorimotor, language, cognitive, emotional) in real-time, as a mirror of the meta-network, resulted in a tailored connectome-based resection which allowed professional musicians to resume their artistic activities following tumor resection: indeed, by preserving crucial networks subserving musical skills, learning and creativity, patients were able to not only to play music again but also to compose new pieces after brain surgery (14). One step forward, Shofty et al. (15) have suggested to use DES in awake patients performing a test of creative thinking (alternate-uses-task). They observed that stimulation at the DMN hubs elicited a reduction of creative fluency, supporting that the DMN is causally linked to creativity. Therefore, DES mapping could be helpful to preserve networks involved in creative cognition during tumor resection, especially in artists.

In summary, creativity is a complex ability mediated by integrated cognitive systems which should be conceived in a multi-demanding, delocalized and constantly-in-motion networking processing. This better understanding of the neurobiology of creative thinking may have important clinical applications, notably for brain surgery. Thus, while neglected for a long time, this unique human ability should be evaluated more regularly in routine practice, particularly in artists.

## Data availability statement

The original contributions presented in the study are included in the article/supplementary material. Further inquiries can be directed to the corresponding author.

## Ethics statement

The studies involving humans were approved by Institutional review board of the ethical comity of research from the French National College of Neurosurgery (N°00011687–2024/07). The studies were conducted in accordance with the local legislation and institutional requirements. The participants provided their written informed consent to participate in this study. Written informed consent was obtained from the individual(s) for the publication of any potentially identifiable images or data included in this article.

## Author contributions

HD: Conceptualization, Data curation, Investigation, Methodology, Supervision, Validation, Writing – original draft, Writing – review & editing.
